# Case Report: Durable therapy response to Osimertinib in rare EGFR Exon 18 mutated NSCLC

**DOI:** 10.3389/fonc.2023.1182391

**Published:** 2023-08-16

**Authors:** Michael Cekay, Philipp F. Arndt, Rio Dumitrascu, Rajkumar Savai, Andreas Braeuninger, Stefan Gattenloehner, Dagmar Steiner, Fritz Roller, Khodr Tello, Katja Hattar, Werner Seeger, Ulf Sibelius, Friedrich Grimminger, Bastian Eul

**Affiliations:** ^1^ Department of Internal Medicine, Justus-Liebig-University Giessen, Universities of Giessen and Marburg Lung Center (UGMLC), Member of the German Center for Lung Research (DZL), Giessen, Germany; ^2^ Max Planck Institute for Heart and Lung Research, Member of the DZL, Member of CPI, Giessen, Germany; ^3^ Institute for Lung Health (ILH), Justus Liebig University, Giessen, Germany; ^4^ Frankfurt Cancer Institute (FCI), Goethe University, Frankfurt, Germany; ^5^ Department of Pathology, Justus-Liebig-University Giessen, Universities of Giessen and Marburg Lung Center (UGMLC), Giessen, Germany; ^6^ Department of Nuclear Medicine, Justus-Liebig-University Giessen, Universities of Giessen and Marburg Lung Center (UGMLC), Giessen, Germany; ^7^ Department of Radiology, Justus-Liebig-University Giessen, Universities of Giessen and Marburg Lung Center (UGMLC), Giessen, Germany

**Keywords:** non-small cell lung cancer, EGFR mutation, tyrosine kinase inhibitors (TKIs), Osimertinib, EGFR Exon 18 insertion

## Abstract

Up to 20% of all non-small cell lung cancer patients harbor tumor specific driver mutations that are effectively treated with tyrosine kinase inhibitors. However, for the rare EGFR deletion-insertion mutation of exon 18, there is very little evidence regarding the effectiveness of tyrosine kinase inhibitors. A particular challenge for clinicians in applying tyrosine kinase inhibitors is not only diagnosing a mutation but also interpreting rare mutations with unclear therapeutic significance. Thus, we present the case of a 65-year-old Caucasian male lung adenocarcinoma patient with an EGFR Exon 18 p.Glu709_Thr710delinsAsp mutation of uncertain therapeutic relevance. This patient initially received two cycles of standard platinum-based chemotherapy without any therapeutic response. After administration of Osimertinib as second line therapy, the patient showed a lasting partial remission for 12 months. Therapy related toxicities were limited to mild thrombocytopenia, which ceased after dose reduction of Osimertinib. To our knowledge, this is the first report of effective treatment of this particular mutation with Osimertinib. Hence, we would like to discuss Osimertinib as a viable treatment option in EGFR Exon 18 p.Glu709_Thr710delinsAsp mutated lung adenocarcinoma.

## 1 Introduction

Lung cancer is a global health problem as it is the most common cause of cancer related deaths worldwide. The prognosis of lung cancer remains poor, as most patients initially present with distant metastases. During the past decades, therapeutic options for lung cancer have improved. Modern treatment of lung cancer relies on multimodal therapeutic concepts and includes radiation, surgery, chemotherapy, immunotherapy and targeted therapies with kinase inhibitors. Patients with driver mutations such as mutations in EGFR, BRAF, ALK, RET, KRAS Gly12Cys, ROS1 and NTRK1/2/3 fusions have most outstandingly benefited from the development of targeted therapies. Yet, these patients represent only a minority (~20%) of the entire lung cancer patient collective (1). A major challenge for oncologists during their day-to-day clinical routine is to determine whether a rare mutational pattern in a non-small cell lung cancer (NSCLC) patient might be responsive to an unapproved tyrosine kinase inhibitor (TKI) therapy (2–4). The scarce clinical evidence available does however show that TKIs such as Afatinib and Osimertinib indeed have clinical efficacy in rare EGFR mutations (5–8). Furthermore, for many mutations it is still unclear if they even have an activating character or if they are mere incidental findings.

## 2 Case presentation

**Figure 1:**
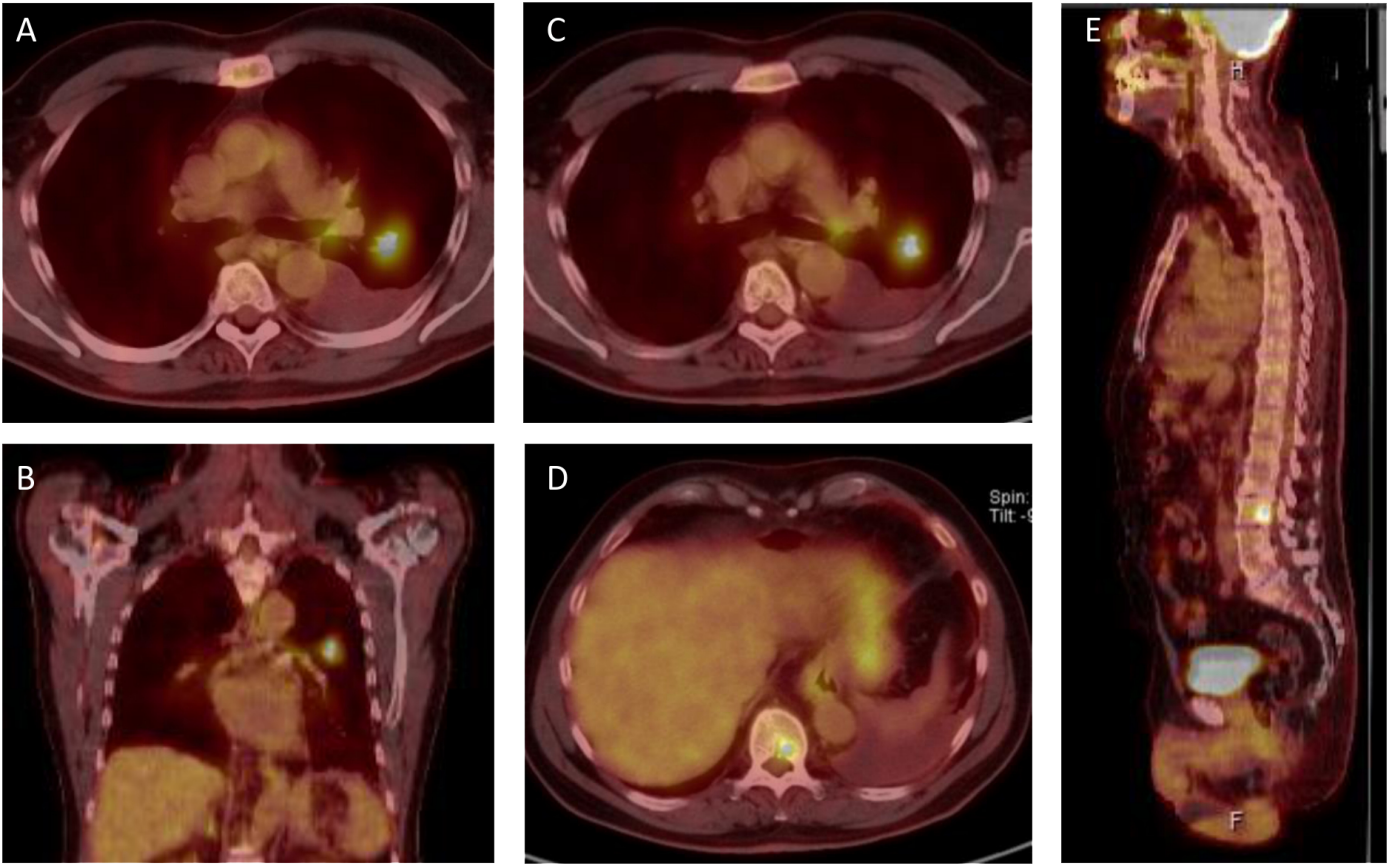
PET-CT scans from initial presentation in october 2021. **(A, B)** The patient presents with a ^18^F-fluourodeoxyglucose (FDG)-positive lesion in the left superior lobe and an ipsilateral pleural effusion. **(C)** ipsilateral FDG positive lymphnode in the aortopulmonary window. **(D, E)** FDG positive bone lesions in the 10^th^ thoracic vertebra **(D)** and the 2^nd^ lumbar vertebra **(E)**, resulting in a clinical classification of T1 N1 M1c, Stage IVB, according to the 8th UICC edition.

**Figure 2:**
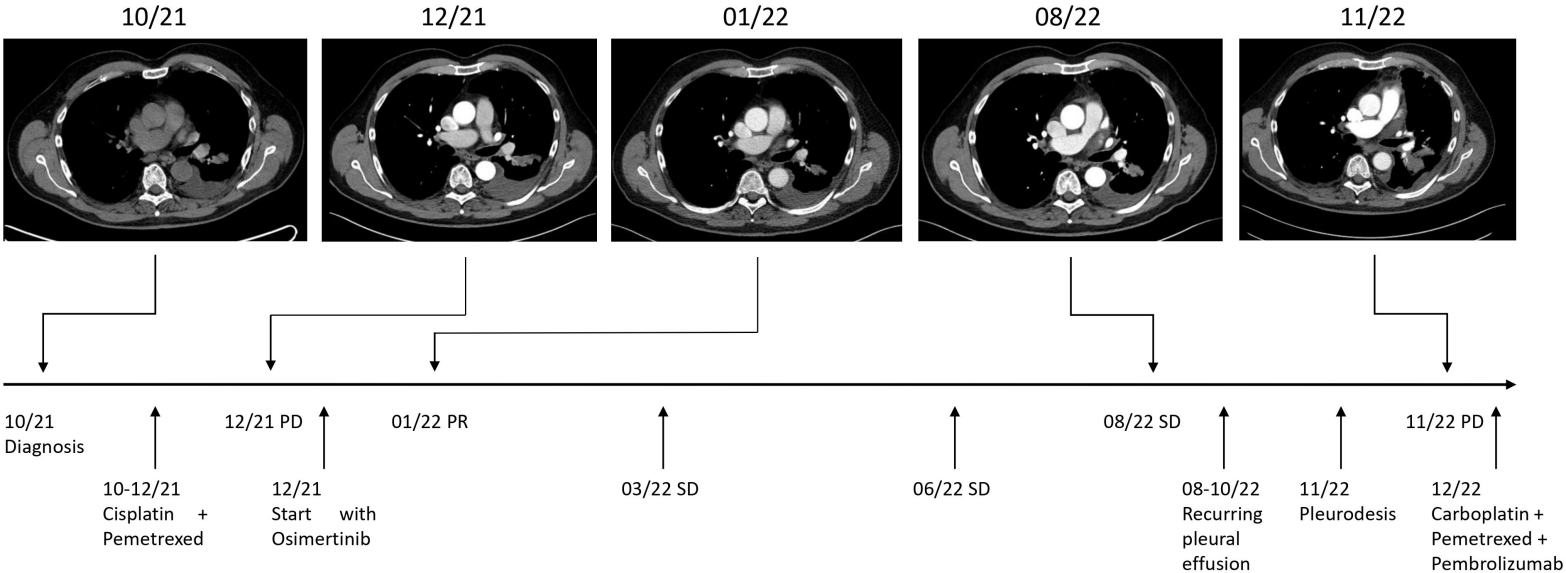
Timeline. Maximum diameters of lesion at diagnosis in 10/21: 15 x 23 mm, at PD (progressive disease) in 12/21: 18 x 30 mm, at PR (partial remission) in 01/22: 14 x 27 mm, and at PD 11/22: 37 x 41 mm. SD (stable disease).

We present the case of a 65-year-old Caucasian male who was diagnosed with stage IV NSCLC in September 2021. The patient initially presented with symptoms of progressive dyspnea, exercise intolerability and recurring thorax pain. As the patient had a known history of cardiovascular disease, cardiac magnetic resonance imaging (MRI) was performed, revealing an incidental nodule of the left posterior inferior lung lobe. He was then referred to our lung cancer center. The patient teaches law as a professor at a university and there was no known family history of cancer. However, the patient presented with 40 pack years of cigarette smoking. The patient did not show any further risk factors for lung cancer such as exposition to asbestos, radiation or other potential hazards. Initial workup included a bronchoscopy with endobronchial ultrasound and transbronchial needle aspiration (EBUS-TBNA). However, two consecutive bronchoscopies failed to deliverer a malignant cytology sample of the tumor for further workup. A subsequent fluorodeoxyglucose positron emission tomography-computed tomography (FDG-PET-CT) revealed a hypermetabolic tumor of the left lung, various bone metastases of the spine and a singular metastasis of the left adrenal gland (**Figure 1**). Finally, the histology of the tumor was obtained through drainage of a pleural effusion of the left lung. Pathological examination revealed an adenocarcinoma of the lung and the initial staging of the patient resulted in cT1 cN1 cM1c and UICC IVB. Comprehensive molecular diagnostics fulfilling standards of the national Network for Genomic Medicine (Germany) were performed. Targeted next generation sequencing (NGS) with a TSO500 panel (Illumina) was performed to detect single nucleotide variants and small insertions or deletions in 523 genes recurrently affected by mutations in various cancer types. This analysis further evaluated copy number variants of 59 genes, microsatellite instability and tumor mutation burden. Additionally, the Archer FusionPlex Lung panel was used to detect fusion transcripts of 17 genes including ALK, ROS1, RET and NTRK1-3. Fluorescence *in situ* hybridization (FISH) was performed to detect MET amplifications. These studies revealed an EGFR Exon 18 mutation (p.Glu709_Thr710delinsAsp), a neomorph U2AF1 mutation and a likely inactivating mutation in PPC6, a negative regulator of MEK. Further, likely and known inactivating mutations in ATM, AR, DDX41 and variants of unknown significance in six further genes were detected (**Table 1**). No ALK, ROS1, RET or NTRK1/2/3 fusion transcripts and no MET amplifications were found. Tumor mutation burden was 8.6 variants/megabase pair (Mbp). At the same time, there was no expression of programmed death-ligand 1 (PD-L1) on tumor cells. After primary diagnosis of the NSCLC in September 2021, we initiated standard of care first-line treatment. The initial regimen was Cisplatin (75 mg/m²) and Pemetrexed (500 mg/m²) administered every three weeks, starting mid October 2021. The patient received two cycles of therapy in total without any major side effects. The bone metastases were additionally treated with intravenous infusions of zolendronic acid every other month, commencing in October 2021. The decision to waive radiation therapy in this patient was based on the absence of significant symptom burden associated with bone metastases such as pain or hypercalcemia. Furthermore, there were no osteolytic lesions at risk of fracturing detectable. The patient exhibited good tolerance to zolendronic acid, which was utilized as an adjunctive therapy alongside all systemic treatments thereafter. To monitor therapeutic success, we conducted a computed tomography (CT) scan in December 2021. Unfortunately, this follow-up scan revealed a progression of the primary tumor according to RECIST criteria. Although none of the distant metastases progressed, the patient’s pleural effusion required more frequent drainage. As the patient furthermore suffered from severe nausea and vomiting from cisplatin, we decided to end chemotherapy and initiate TKI-therapy with Osimertinib. This decision was based on case reports previously describing the use of TKIs for this particular EGFR mutation with variable success (9–12). We began treatment with Osimertinib at the beginning of December 2021, starting with 80 mg taken orally once daily. The patient tolerated the administration of Osimertinib well and did not have any clinical signs of side effects or toxicities at first follow-up. Nevertheless, it was necessary to reduce Osimertinib dosing to 40 mg daily as the patient developed worsening thrombocytopenia (nadir of 114 giga/l) three weeks in to his TKI treatment. After dose reduction, the thrombocyte count remained stable at >120 giga/l. We conducted a short-term CT follow-up examination in January 2022, which revealed comprehensive therapeutic response of the NSCLC to Osimertinib therapy. The various bone metastases displayed increasing sclerosis compatible with a notable therapeutic response. The aforementioned pleural effusion likewise regressed. Additionally, the patient continued to tolerate Osimertinib without any further notable toxicities. Follow-up CT scans were conducted in March and August of 2022, which showed stable disease based on Response Evaluation Criteria in Solid Tumors (RECIST). However, the patient again developed a progressive pleural effusion in August 2022. The effusion was initially solely monitored using ultrasound. Regrettably, tumor progression was eventually noted on a further follow-up CT scan in November 2022 and Osimertinib therapy was discontinued. The pleural effusion was now treated with pleurodesis. Again, malignant NSCLC cells were detectable in the pleural fluid and we repeated a comprehensive pathological and molecular workup using NGS (TSO500). Here, PD-L1 status could be assessed to 5% on tumor cells in the newly acquired sample but otherwise the mutation pattern was identical to the initial analysis we conducted. As the patient reported a history of 40 pack years and no prior treatment with immunotherapy, the decision was made for a chemo-immunotherapy re-induction third line therapy regimen. The therapy was initiated in late November 2022 and consisted of Carboplatin AUC 5 (550mg absolute dose), Pemetrexed 500 mg/m² and Pembrolizumab 200mg administered every three weeks (**Figure 2**). The patient received two cycles of this treatment and tolerated it well. In January 2023, a follow-up CT scan revealed a mixed response to the applied cycles of chemo-immunotherapy. The patient again exhibited progressive pleural effusions on both sides, while the primary tumor in the left lung remained constant. The size of mediastinal lymph nodes was decreasing, but some lymph nodes in the retroperitoneal and mediastinal regions showed minor progression. At the same time, bone lesions remained stable compared to previous CT scans and no further distant metastases were detectable. The CT examination was classified as stable disease based on RECIST. As the patient consistently showed good therapy tolerance, two additional cycles of chemo-immunotherapy were administered with unchanged dosage. In February 2023, another follow-up CT scan yet again showed a stable disease state based on RECIST. Both the primary lung tumor and lymph nodes displayed no significant changes in size. Notably, the bone metastases demonstrated progressive sclerosis further indicating therapy response. After completing four cycles of chemo-immunotherapy, Carboplatin and Pemetrexed were discontinued, while Pembrolizumab monotherapy was continued every three weeks. As of May 2023, the patient underwent another CT follow-up examination, which once more showed a stable disease state with a minor reduction of primary lung tumor size. No new distant metastases or other irregularities were observed. As of June 2023, the patient is currently continuing Pembrolizumab monotherapy.

**Table 1:** Genetic alterations detected.

Gens	Coding change	Protein change	VAF %	Functional impact
EPHA5	c.560A>G	p.Asp187Gly	34	unknown
DDX41	c.618del	p.Ile207PhefsTer15	51	likely inactivating
EGFR	c.2127_2129del	p.Glu709_Thr710delinsAsp	39	known activating
MET	c.1958C>T	p.Ser653Phe	40	unknown
PTPRD	c.3325C>T	p.Arg1109Cys	32	unknown
PPP6C	c.306del	p.Phe102LeufsTer19	13	likely inactivating
ARID5B	c.1490T>C	p.Ile497Thr	51	unknown
ATM	c.6115G>A	p.Glu2039Lys	61	known inactivating
ATM	c.9164G>C	p.Trp3055Ser	25	unknown
U2AF1	c.101C>T	p.Ser34Phe	36	known activating
AMER1	c.1143_1145del	p.Glu387del	90	unknown
AR	c.2453del	p.Pro818GlnfsTer5	72	likely inactivating

VAF, variant allele frequency.

## 3 Discussion and conclusion

For patients with classical EGFR mutations in exon 19 and 21 TKI therapies have had a remarkable impact on progression free and overall survival. The optimal therapy for rare EGFR Exon 18 p.Glu709_Thr710delinsAsp mutated NSCLC patients has yet not been determined. This case report emphasizes the importance of sophisticated genetic testing via NGS in NSCLC patients. Unfortunately, not all lung cancer patients receive NGS prior to therapy initiation. At the same time, growing knowledge and therapeutic possibilities with newly developed TKIs are becoming more challenging for clinicians as it becomes increasingly complex to make ideal treatment decisions. This is especially true for applying targeted therapies in rare mutations with unclear or yet unknown clinical implication. For this reason, various databases have been developed during recent years to catalog available knowledge on rare mutations and support clinicians in making appropriate treatment decisions. However, it is also imperative for clinicians to share new insights in applying targeted therapies. Numbers of patients with uncommon mutations will continue to be considerably low and structured clinical trials for such mutations will likely remain rare. Therefore, case reports may offer valuable insight for such mutations. Additionally, structured large-scale national or international investigations such as by the national network for genomic medicine in Germany or the French ERMETIC-IFCT network (13, 14) are even more important. Regarding this clinical case, it is notable that we conducted a second NGS examination with pleural fluid obtained at tumor progression in November 2022. This examination revealed an identical mutation pattern in comparison to the assay we conducted at first diagnosis. The only marker differing from the initial workup was PD-L1. Nevertheless, the tumor progressed regardless of Osimertinib therapy. Several previous case reports have been published describing the use of TKI-therapy in NSCLC patients with rare EGFR mutations. However, the effectiveness of these treatments have often been limited. Moreover, all patients in these reports received either first- or second-generation EGFR-TKIs and their clinical characteristics and demographics differed significantly from the patient described in this report. Ackermann et al. (15) presented the case of an 88-year-old female non-smoker who received Erlotinib and exhibited a partial response lasting for 4 months. Sousa et al. (10) described the case of a 66-year-old female with a smoking history who was treated with Gefitinib and showed a progression-free survival of 4 months and an overall survival of 24 months. Furthermore, Xu et al. (4) conducted an analysis of Chinese patients with various rare EGFR mutations, comparing the effectiveness of first or second generation EGFR-TKIs to chemotherapy or a combination of chemotherapy and TKIs. This study suggested that a combination of first generation TKIs and chemotherapy could be equally effective as treatment with Afatinib as a second generation TKI alone. Additionally, Wei et al. (12) reported successful treatment of EGFR Exon 18 Insertion p.Glu709_Thr710delinsAsp mutated NSCLC with Afatinib, followed by Almonertinib after tumor progression. The progression-free survival for Afatinib was 23 months, which was nearly twice as long as in our reported case. However, the patient in this report had different demographics and clinical characteristics, including Asian ethnicity and different tumor stage. Previously both Osimertinib and Afatinib have shown efficacy in clinical trials with rare EGFR mutations. During the LUX Lung trials, patients treated with Afatinib showed an overall response rate (ORR) of up to 70% whereas patients in the UNICORN study treated with Osimertinib showed an ORR of 60% (5, 6, 8). However, none of the patients included carried an EGFR Exon 18 Insertion p.Glu709_Thr710delinsAsp mutation making it yet unclear to judge which TKI provides the greatest therapeutic benefit for this particular mutation. However, as NSCLC commonly metastasizes to the brain, we decided to implement Osimertinib instead of Afatinib due to its superior intracerebral efficacy. Our decision was furthermore based on its more favorable profile regarding adverse effects. Additionally, the use of immunotherapy as initial treatment for this patient is similarly debatable. Our decision was to refrain from administering immunotherapy as first line treatment due to the identified EGFR mutation of uncertain clinical significance. In addition, the absence of PD-L1 expression in the tumor cells of the pleural effusion likewise influenced our decision. However, the extent to which these tumor cells from the pleural fluid resemble the primary NSCLC lung tumor remains likewise debatable. Nonetheless, initiating immunotherapy upfront would have been justifiable in this case, given the patients smoking history of 40 pack years. Another point of discussion revolves around the re-induction therapy regimen following treatment failure of Osimertinib. Applying the IMPOWER150 regimen, comprising of Carboplatin, Paclitaxel, Bevacizumab and Atezolizumab would have also been a viable therapeutic option. In conclusion, the optimal treatment approach for this particular mutation remains undecided and might also depend on individual patient characteristics. To our knowledge, this is the first description of a successful therapeutic response for Osimertinib treatment in an EGFR Exon 18 p.Glu7-09_Thr710delinsAsp mutated NSCLC patient. This case report contributes to the understanding of this rare mutation and we would like to propose Osimertinib as a feasible treatment option.

## Data availability statement

The original contributions presented in the study are included in the article/supplementary material. Further inquiries can be directed to the corresponding author.

## Ethics statement

Ethical review and approval was not required for the study on human participants in accordance with the local legislation and institutional requirements. The patients/participants provided their written informed consent to participate in this study. Written informed consent was obtained from the individual(s) for the publication of any potentially identifiable images or data included in this article. The authors are accountable for all aspects of this work. Tissues were used in accordance with the ethics committee of the University of Giessen and German center for lung research.

## Author contributions

BE, WS and FG initiated and supervised the study. MC, AB and PA collected data, and drafted the manuscript. BE, AB, SG, RS, WS and FG revised the manuscript for important intellectual content. All authors reviewed and edited the manuscript.

## References

[1] Altorki NK Markowitz GJ Gao D Port JL Saxena A Stiles B. The lung microenvironment: an important regulator of tumour growth and metastasis. Nat Rev Cancer (2019) 19(1):9–31. doi: 10.1038/s41568-018-0081-9 30532012 PMC6749995

[2] Wu JY Yu CJ Chang YC Yang CH Shih JY Yang PC. Effectiveness of tyrosine kinase inhibitors on "uncommon" epidermal growth factor receptor mutations of unknown clinical significance in non-small cell lung cancer. Clin Cancer Res (2011) 17(11):3812–21. doi: 10.1158/1078-0432.CCR-10-3408 21531810

[3] Wu SG Chang YL Lin JW Wu CT Chen HY Tsai MF. Including total EGFR staining in scoring improves EGFR mutations detection by mutation-specific antibodies and EGFR TKIs response prediction. PloS One (2011) 6(8):e23303. doi: 10.1371/journal.pone.0023303 21858063 PMC3153495

[4] Xu H Yang G Li W Li J Hao X Xing P. EGFR Exon 18 mutations in advanced non-small cell lung cancer: a real-world study on diverse treatment patterns and clinical outcomes. Front Oncol (2021) 11:713483. doi: 10.3389/fonc.2021.713483 34540680 PMC8445032

[5] Bar J Peled N Schokrpur S Wolner M Rotem O Girard N. UNcommon EGFR mutations: international case series on efficacy of Osimertinib in real-life practice in first-LiNe setting (UNICORN). J Thorac Oncol (2023) 18(2):169–80. doi: 10.1016/j.jtho.2022.10.004 36307041

[6] Cho JH Lim SH An HJ Kim KH Park KU Kang EJ. Osimertinib for patients with non-small-cell lung cancer harboring uncommon EGFR mutations: a multicenter, open-label, phase II trial (KCSG-LU15-09). J Clin Oncol (2020) 38(5):488–95. doi: 10.1200/JCO.19.00931 PMC7098834 31825714

[7] Yang JC Schuler M Popat S Miura S Heeke S Park K. Afatinib for the treatment of NSCLC harboring uncommon EGFR mutations: a database of 693 cases. J Thorac Oncol (2020) 15(5):803–15. doi: 10.1016/j.jtho.2019.12.126 31931137

[8] Yang JC Sequist LV Geater SL Tsai CM Mok TS Schuler M. Clinical activity of afatinib in patients with advanced non-small-cell lung cancer harbouring uncommon EGFR mutations: a combined post-hoc analysis of LUX-Lung 2, LUX-Lung 3, and LUX-Lung 6. Lancet Oncol (2015) 16(7):830–8. doi: 10.1016/S1470-2045(15)00026-1 26051236

[9] Klughammer B Brugger W Cappuzzo F Ciuleanu T Mok T Reck M. Examining treatment outcomes with erlotinib in patients with advanced non-small cell lung cancer whose tumors harbor uncommon EGFR mutations. J Thorac Oncol (2016) 11(4):545–55. doi: 10.1016/j.jtho.2015.12.107 26773740

[10] Sousa AC Silveira C Janeiro A Malveiro S Oliveira AR Felizardo M. Detection of rare and novel EGFR mutations in NSCLC patients: Implications for treatment-decision. Lung Cancer (2020) 139:35–40. doi: 10.1016/j.lungcan.2019.10.030 31715539

[11] Van Acker L Stevens D Vermaelen K Surmont V. Afatinib for the treatment of advanced non-small-cell lung cancer harboring an epidermal growth factor receptor exon 18 E709_T710delinsD mutation: a case report. J Med Case Rep (2021) 15(1):562. doi: 10.1186/s13256-021-02994-0 34809713 PMC8609878

[12] Wei Y Cui Y Guo Y Li L Zeng L. A lung adenocarcinoma patient with a rare EGFR E709_T710delinsD mutation showed a good response to Afatinib treatment: A case report and literature review. Front Oncol (2021) 11:700345. doi: 10.3389/fonc.2021.700345 34178699 PMC8226096

[13] Beau-Faller M Prim N Ruppert AM Nanni-Metéllus I Lacave R Lacroix L. Rare EGFR exon 18 and exon 20 mutations in non-small-cell lung cancer on 10 117 patients: a multicentre observational study by the French ERMETIC-IFCT network. Ann Oncol (2014) 25(1):126–31. doi: 10.1093/annonc/mdt418 PMC3868323 24285021

[14] Ng PK Li J Jeong KJ Shao S Chen H Tsang YH. Systematic functional annotation of somatic mutations in cancer. Cancer Cell (2018) 33(3):450–62.e10. doi: 10.1016/j.ccell.2018.01.021 29533785 PMC5926201

[15] Ackerman A Goldstein MA Kobayashi S Costa DB. EGFR delE709_T710insD: a rare but potentially EGFR inhibitor responsive mutation in non-small-cell lung cancer. J Thorac Oncol (2012) 7(10):e19–20. doi: 10.1097/JTO.0b013e3182635ab4 PMC3444741 22982663

